# The Intersection of Dermatological Dilemmas and Endocrinological Complexities: Understanding Necrobiosis Lipoidica—A Comprehensive Review

**DOI:** 10.3390/biomedicines12020337

**Published:** 2024-02-01

**Authors:** Corina Ionescu, Aida Petca, Mihai Cristian Dumitrașcu, Răzvan-Cosmin Petca, Andreea Iuliana Ionescu (Miron), Florica Șandru

**Affiliations:** 1 Department of Dermatovenerology, “Carol Davila” University of Medicine and Pharmacy, 020021 Bucharest, Romania; corina.ionescu@rez.umfcd.ro (C.I.); florica.sandru@umfcd.ro (F.Ș.); 2 Dermatology Department, “Elias” University Emergency Hospital, 011461 Bucharest, Romania; 3 Department of Obstetrics and Gynecology, “Carol Davila” University of Medicine and Pharmacy, 020021 Bucharest, Romania; aida.petca@umfcd.ro (A.P.); mihai.dumitrascu@umfcd.ro (M.C.D.); 4 Department of Obstetrics and Gynecology, Elias Emergency University Hospital, 011461 Bucharest, Romania; 5 Department of Obstetrics and Gynecology, University Emergency Hospital of Bucharest, 050098 Bucharest, Romania; 6 Department of Urology, “Carol Davila” University of Medicine and Pharmacy, 020021 Bucharest, Romania; 7 Department of Urology, “Prof. Dr. Th. Burghele” Clinical Hospital, 050659 Bucharest, Romania; 8 Department of Oncological Radiotherapy and Medical Imaging, “Carol Davila” University of Medicine and Pharmacy, 020021 Bucharest, Romania; andreea-iuliana.miron@drd.umfcd.ro; 9 Department of Medical Oncology, Colțea Clinical Hospital, 030167 Bucharest, Romania

**Keywords:** necrobiosis lipoidica, diabetes mellitus, thyroid disease, metabolic syndrome

## Abstract

Background: Necrobiosis lipoidica (NL) is a rare granulomatous skin disorder with a predilection for females, often associated with diabetes mellitus (DM). This paper aims to comprehensively review the literature on NL, focusing on its association with DM, thyroid disorders, and the metabolic syndrome. Methods: A systematic search was conducted in English-language literature from inception to October 2023, utilizing PubMed. We identified 530 studies and selected 19 based on clinical significance, statistical support, and relevance to the paper’s goals. Results: The coexistence of NL and DM is prevalent, with rates ranging from 11% to 65.71%. NL may precede DM diagnosis and a correlation between NL and increased daily insulin requirements has been observed in such patients. NL is suggested as a potential prognostic marker for DM complications; however, recent studies question this association, highlighting the need for further research. Studies in the context of NL and Thyroid Disease indicate a correlation, especially with autoimmune thyroiditis. Regarding NL and Metabolic Syndrome, the prevalence of metabolic syndrome among NL patients is notably higher than in the general population. Additionally, DM patients with ulcerated NL commonly exhibit hypertension or obesity, raising questions about the potential influence of hypertension and obesity on NL ulcerations. Conclusion: Additional research is required to untangle the complex connections between NL and various comorbidities.

## 1. Introduction

Necrobiosis lipoidica (NL) is a relatively rare, chronic, granulomatous skin disorder characterized by deterioration of collagen, granuloma formation, fat deposition, and thickening of blood vessel walls [1]. NL is characterized by greater rates among females, with a female-to-male ratio of 3:1. For type 1 diabetics, the age of onset is usually in the third decade of life; for type 2 diabetics and nondiabetics, the age of onset is in the fourth decade [2,3].

Lesions associated with NL generally manifest as one to three well-defined painless papules or nodules. They exhibit active erythematous borders and gradually merge, forming oval-shaped plaques. The plaques exhibit a violaceous hue and feature a core region that first presents as reddish-brownish, but subsequently advances to a yellowish-brownish discoloration [2,4]. The central region frequently exhibits characteristics such as atrophy, waxiness and erosion of the skin. Telangiectasis can occur as a consequence of collagen deterioration taking place beneath the epidermal layer [2]. The Koebner phenomenon can also occur. As lesions progress in their development, they become less active and more atrophic, which leaves them more vulnerable to trauma-induced ulceration [1]. About 30% of patients develop ulcerations and become painful [5]. Although lesions can occasionally be observed on the face, scalp, torso, groin and upper extremities, NL usually limits itself to the lower limbs, bilaterally. Diagnosis requires the comprehensive integration of clinical findings and histological examination. Histological observations commonly reveal a pattern of palisaded and interstitial granulomatous dermatitis, accompanied by localized degeneration of connective tissue and vascular alterations involving the dermis and the subcutaneous tissue. Described as a “sandwich-like” configuration, the palisaded granulomas are placed in a horizontal layer with intermixed layers of altered, homogenized, “necrobiotic” collagen. However, it is important to note that the clinical features may exhibit variations during the course of the disease and across diverse individuals [6]. The findings of a retrospective investigation, including 97 patients with NL, indicate the presence of histopathologic variations between cases associated with diabetes and those without diabetes [6]. The proportion of epidermal acanthosis and neutrophils was higher among individuals diagnosed with diabetes, while patients without diabetes exhibited a higher prevalence of sarcoid and tuberculoid granulomas and eosinophils [6]. In particular cases, there may be lymphocytic aggregates, sometimes with germinal centers, found in the deep dermis or subcutaneous tissue [7]. Another unusual histopathological finding might be the presence of mucin, which has been linked to NL associated with autoimmune thyroiditis [8].

Following diagnosis, it is critical to keep monitoring for the possible occurrence of ulcerations and treat it aggressively in order to reduce the risk of development into squamous cell carcinoma [9,10,11,12,13].

The management of NL presents significant difficulties; even when lesions undergo resolution, post-inflammatory and atrophic alterations might persist. The recalcitrant nature of this disease greatly impacts the quality of life of affected individuals [14]. Steroids, namely topical, intralesional, or sometimes systemic, currently serve as the primary therapeutic approach [15,16]. Additional therapeutic interventions that have been used encompass topical tacrolimus, PUVA, antimalarials, photodynamic therapy, fumaric acid esters, pentoxifylline, ticlopidine, biologics, cyclosporine, and surgery with excision followed by skin grafting [15,17,18,19,20,21,22,23,24,25,26].

The etiology and pathophysiology of NL remain uncertain. The etiological proposals about microangiopathy are more widespread due to its higher frequency in diabetic patients, especially those who require insulin [2].

The occurrence of NL is often observed in conjunction with diabetes mellitus, leading to the previous designation of this condition as “necrobiosis lipoidica diabeticorum”. The acknowledgment that NL can manifest even in the absence of diabetes prompted a modification in the nomenclature [27]. The condition has been otherwise observed in individuals diagnosed with sarcoidosis, inflammatory bowel disease, autoimmune thyroiditis, rheumatoid arthritis, monoclonal gammopathy, as well as in otherwise healthy individuals with normal glucose metabolism and no prior history of autoimmune disorders [28,29,30].

The association between NL and diabetes mellitus has been the subject of extensive research and analysis. Since Oppenheim’s 1929 description of the condition, numerous investigations have revealed that over 60% of people have diabetes at the time of NL diagnosis [31]. In contrast, it is estimated that NL occurs in only around 3% of individuals throughout the progression of DM [32].

DM is a widespread health issue of global significance, with a projected worldwide prevalence of 9.3% in 2019 [33]. Approximately 529 million people worldwide were estimated to suffer from DM in 2021 [34]. Furthermore, between 40% and 50% of the global population is classified as “high risk” (i.e., prediabetes) [35]. Findings from a study based on data derived from the National Health Interview Survey, conducted in 2016 and 2017, estimate the prevalence of adults in the US diagnosed with type 2 diabetes (T2DM) to stand at 8.5% [36]. In 2022, the Center for Disease Control and Prevention Diabetes Surveillance System and other national databases documented a prevalence rate of diagnosed diabetes among adults at approximately 11.3% [37]. T2DM constitutes roughly 90% of the total cases of diabetes [27,33]. Globally, an estimated 46.5% of T2DM cases remain undiagnosed [38]. The available global data indicates that the prevalence of T2DM among individuals aged from 15 to 39 years has increased from 117 to 183 per 100 000 persons, between the years 1990 and 2019 [39].

Since 1990, the global incidence of DM has more than doubled, and is expected to rise more [40]. There is a concern that the prevalence of DM will continue to grow significantly in light of the notable rise in childhood obesity [39]. Between 21% and 33% of Americans are expected to have the disease by 2050 [41]. Moreover, the global incidence, death, and disability-adjusted life-years (DALYs) related to DM in the year 2017 were recorded as 22.9 million, 1.37 million, and 67.9 million, respectively. Projections indicate that these figures will increase to 26.6 million, 1.59 million, and 79.3 million by the year 2025 [40].

Diabetes has a detrimental effect on quality of life and raises the risk of all causes of death by two to three times [40]. DM is projected to receive a greater healthcare resource allocation than any other medical condition [42]. There are multiple factors, apart from complications associated with diabetes, that contribute to the influence of DM on both quality of life and cost of healthcare. The condition is commonly linked to a significant occurrence of depression, and it has negative effects on employment, absenteeism, and work productivity [43,44]. Despite significantly contributing to mortality, morbidity, and healthcare costs, DM poses a substantial challenge to the world’s health and progress [38].

Elevated levels of serum glucose have been observed to have harmful effects on several cell types, such as endothelial cells, neurons, renal cells, keratinocytes, and fibroblasts [45,46]. Skin conditions are present in around one-third of diabetics and often develop prior to diagnosis, making them crucial in the early detection of underlying illness [45]. Moreover, it has been suggested that patients may be more motivated to improve their DM control when their cutaneous condition is apparent [47]. Both non-infectious and infectious conditions have been identified as dermatologic complications of DM. Furthermore, skin complications might arise from diabetic neuropathy and angiopathy. Pruritus, acanthosis nigricans, necrobiosis lipoidica, scleredema adultorum of Buschke, stiff hand syndrome, Huntley’s papules, and bullous diabeticorm are several examples of documented non-infectious skin diseases [48,49,50,51,52,53,54,55,56]. Skin infections caused by bacteria and fungi are more common in diabetics. Diabetic foot syndrome and diabetic dermopathy can be attributed to the presence of diabetic neuropathy and angiopathy. Moreover, cutaneous side effects may be triggered by antidiabetic therapy [45]. The administration of insulin has been observed to elicit localized reactions, including lipohypertrophy, lipoatrophy, and both immediate and delayed allergic reactions [45].

Although the most common comorbidity mentioned in the literature among individuals affected by NL is DM, metabolic syndrome and thyroid disorders have gained more attention lately. Thus, this study aims to thoroughly analyze the literature about the association of NL and different comorbidities, with a particular focus on DM and to provide a brief overview of its etiopathogeneses and current treatment barriers.

## 2. Materials and Methods

**Figure 1:**
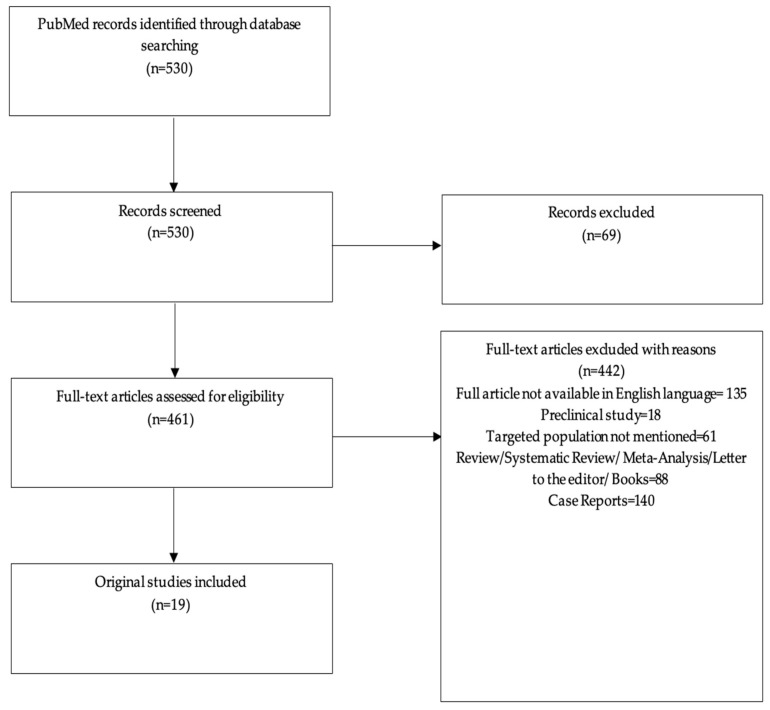
PRISMA flow diagram of the screening process.

This paper is a comprehensive review of the literature published in English from inception to October 2023. For this purpose, a comprehensive search was conducted on the PubMed database (www.pubmed.gov), accessed on 14 October 2023, utilizing two specific keywords: “necrobiosis lipoidica” and “diabetes”. We identified a total of 530 studies, which were subsequently searched manually to meet our study’s goals. Additionally, we excluded animal studies, studies with poor methodology, case reports, meta-analyses, and reviews. The selection process resulted in a total of 19 human studies that were clinically significant and novel, featuring a range of statistical support at different levels. The included articles were evaluated for relevant cross-references. A PRISMA flow diagram was created in order to summarize the screening process visually (Figure 1).

## 3. Results

### 3.1. Necrobiosis Lipoidica and Diabetes Mellitus

The coexistence of type 1 diabetes (T1DM) or T2DM with NL is frequent among patients, albeit not always seen [57,58,59]. The prevalence rates of diabetes in this particular population have been found to range significantly from 11 to 65.71 percent [59,60].

A recent German retrospective study assessed the coexistence of comorbidities in 98 patients with NL. It was discovered that 53 patients (54%) had a medical history of diabetes; interestingly, five of these patients experienced the onset of diabetic symptoms subsequent to their first diagnosis of NL (Table 1). Out of the 47 cases, T1DM was found in 22 (22.4% of all NL patients) and T2DM in 25 (25.5% of all NL patients). Out of all diabetic NL patients, women made up 79% (N = 33) [61]. Erfurt-Berge evaluated the association of NL with other comorbidities over time, adding two supplementary retrospective studies to the above statements. According to a 2015 multicenter-study including 100 patients, 43 patients (43%) had a medical history of diabetes, with 34.9% having T1DM and 65.2% having T2DM, with a ratio of 1:2. The proportion of female patients among those diagnosed with diabetes was found to be 72.1% [62] (Table 1). A 2012 study involving 52 patients also had similar findings. In their medical history, 24 patients (46%) had DM; of these, 16% had T1DM, and 31% had T2DM, again with a 1:2 ratio. Women made up 66% of these patients [63].

Nevertheless, NL is a very uncommon condition among patients suffering from DM. The frequency of NL in diabetic patients ranges from 0.3% to 2.8% [59,64,65]. In a study conducted in Scotland, healthcare clinicians responsible for the care of children with diabetes (aged under 15) were surveyed regarding skin disorders. The results revealed that out of a total of 1557 children, only one child (0.06 percent) received a confirmed diagnosis of NL [66].

NL has been observed to potentially occur prior to the diagnosis of DM. A retrospective study was conducted on a group of 65 patients diagnosed with NL, wherein 16 percent of them either met the criteria for diabetes or exhibited impaired glucose tolerance. Subsequently, a five-year follow-up was conducted on a subset of 42 patients who initially did not have diabetes and had normal glucose tolerance. The findings indicated that within this subset, 7% developed either diabetes or abnormal glucose tolerance within the five-year period [59]. Nonetheless, in Marcoval et al.’s 35-patient study, the onset of DM occurred a mean of 135.70 months before the onset of NL in 20 patients. Three patients received the diagnoses of DM and NL simultaneously. During the course of the follow-up, none of the initially DM-free patients developed the disease [60] (Table 1).

#### 3.1.1. Necrobiosis Lipoidica, a Prognostic Marker in Diabetes Mellitus?

It is uncertain whether NL is a prognosis and severity marker. A multicenter retrospective study including 64,133 patients with T1DM found a correlation between increased daily insulin requirements and NL [67] (Table 2).

Two small case-control studies suggest that both pediatric and adult patients diagnosed with NL and DM are susceptible to an increased likelihood of developing nephropathy and retinopathy. They demonstrate the fact that the presence of NL could serve as an indicator for the occurrence of nephropathy and retinopathy [68,69]. A. Verotti et al. found that out of 18 patients diagnosed with T1DM and NL, 12 (66.6%) developed retinopathy vs. 4 (10.0%) from the control group (*p* < 0.001) (Table 2). The study also proved that 10 (55.5%) patients diagnosed with T1DM as well as with NL had microalbuminuria vs. 5 (12.5%) patients from the control group (*p* < 0.001) [68] (Table 2). W F Kelly et al. had similar findings [69]. Neither studies identified a correlation between NL and peripheral neuropathy. Moreover, Trihan et al.’s cross-sectional study included 213 DM-positive subjects, supporting the association between microvascular involvement and NL. An increased risk (OR 9.7, *p* ≤ 0.001) of presenting microvascular disease (retinal or nephrological) in individuals with NL compared to those without NL was reported in the study [65] (Table 2).

Nonetheless, the biggest and most recent study on this topic did not confirm the association between NL and the development of retinopathy or nephropathy. Hammer et al. found that a concurrent occurrence of retinopathy and NL was observed in 2.0% of patients diagnosed with T1DM. A total of 1.1% of the patients with T1DM who did not have NL were found to have retinopathy. The statistical analysis indicated that this difference was not significant (*p* = 0.092) [67] (Table 2).

#### 3.1.2. The Relationship between Necrobiosis Lipoidica and Glycemic Control

The management of glycemic levels may potentially improve the prognosis of NL in individuals diagnosed with DM. However, currently, there is a limited availability of literature that provides a comprehensive overview of the association between glycemic control in individuals with DM and the prognosis of NL.

According to the findings of a retrospective study, NL lesions are more prone to resolution subsequent to pancreas transplantation compared to undergoing kidney transplantation alone [70]. The study included 15 patients: group I (N = 11) consisted of patients who underwent pancreas-only or pancreas-plus-kidney transplants, and group II (N = 4) included patients who underwent kidney-only transplants (Table 3). Out of the eleven patients in group I, five exhibited NL prior to the transplantation procedure, and all of them successfully experienced the clearance of these lesions. A patient had an NL recurrence linked to transplant rejection. Within the second group, it was observed that a single patient who exhibited NL prior to the transplantation procedure continued to have persistent lesions subsequent to the transplant. Thus, resolution of NL was noted in 45% (5 out of 11) of the cases in the group of patients who underwent pancreas-only or pancreas +/− kidney transplantation. However, the one patient who had active NL and underwent a kidney transplant did not show any amelioration of the lesion subsequent to the intervention [70] (Table 3).

Furthermore, in the Ryan et al. study, out of the 12 diabetic type 1 patients who successfully underwent islet transplantation, the one patient who suffered from NL reported the amelioration of the lesions [71] (Table 3).

In a 1993 case-control study including 20 patients with DM, individuals with NL exhibited elevated levels of glycosylated hemoglobin compared to control participants (8.6 ± 1.5 (mean ± SD) vs. 7.4 ± 1.7, *p* = 0.02) [69] (Table 3).

### 3.2. Necrobiosis Lipoidica and Thyroid Disease

The results of multiple studies indicate a possible correlation between necrobiosis lipoidica and thyroid disease. Erfurt-Berge et al. identifies a prevalence of thyroid disease that is three times higher than the overall occurrence, which they estimated to be 5.5% [61] (Table 1). This is also demonstrated in their previous analyses and is statistically significant (*p* < 0.05) [61,62,63]. In their 2022 retrospective study involving 98 patients, it was found that 19 cases (19.4%) had thyroidal function disorders, with 7 cases of autoimmune origin (6 Hashimoto thyroiditis, 1 Graves disease) (Table 1). A total of six female individuals (6.1%) were found to have both ulcerations and thyroid function disease, while no reports of this co-occurrence were observed among males. Notably, in their series of studies, 36.8% of all thyroidal dysfunctions reported had an autoimmune origin [61] (Table 1).

The findings of two extensive retrospective studies conducted in the United States indicate that thyroid disease is observed in around 30% to 40% of the patients with concurrent DM and NL, as well as in around 24.5%,and 35.7%, respectively, of the total patients affected by NL only. However, these findings demonstrate a lack of relevance (*p* = 0.4; *p* = 0.466) [72,73] (Table 1). 

Additional evidence of a correlation with thyroid disease was observed in a comprehensive retrospective study, wherein it was shown that 24.8% of patients with both T1DM and NL exhibited increased levels of thyroid antibodies. In contrast, only 17.5% of those with T1DM alone had such increased antibody levels. However, the *p*-value did not reach statistical significance (*p* = 0.051) (Table 1).

Furthermore, it was observed that patients diagnosed with both NL and T1DM had a higher overall susceptibility to celiac disease in comparison to those with T1DM alone. The prevalence of celiac disease was found to be 3.4% among patients with both conditions, whereas it was only 1% among patients with type T1DM (*p* = 0.0035) [67] (Table 1).

Thus, further research is required to validate the correlation between NL and thyroid disease or celiac disease.

### 3.3. Necrobiosis Lipoidica and Metabolic Syndrome

The presence of comorbidities is frequently observed in individuals diagnosed with NL. Apart from DM, NL patients have also been documented to have dyslipidemia, obesity, hypertension, coronary artery disease, stroke, and reduced renal function. The second most frequent secondary diagnosis among NL patients was arterial hypertension.

Severon et al.’s study findings indicate a higher prevalence of hypertension among patients with T1DM or T2DM and NL, with rates of 49% and 77%, respectively, compared to the estimated prevalence in the general population with T1DM or T2DM (30% and 60%) (*p* = 0.003) (Table 1). It is not unexpected that there were notably elevated rates of hypertension (*p* < 0.001), hyperlipidemia (*p <* 0.001), obesity (*p* < 0.001), and coronary artery disease (*p* = 0.008) among patients with T2DM in comparison to those with T1DM (Table 1). Nevertheless, the incidence of myocardial infarction and stroke in patients with T2DM and NL was shown to be greater compared to the global population affected only by T2MD [73].

Furthermore, a previous study has reported a heightened prevalence of metabolic syndrome among individuals diagnosed with NL. Hashemi et al. highlights the association between obesity and NL, which was observed at comparable rates in individuals with and without diabetes (52.2% vs. 50.7%) (Table 1). This suggests that metabolic variables other than blood glucose may play a role in the pathophysiologic features of this condition. Thus, the risk factors mentioned above are more colinear than independent [72].

In a German retrospective study conducted on 262 patients affected by NL, a total of 132 cases fulfilled the metabolic syndrome criteria [74] (Table 1).

Erfurt-Berge et al. also reported quite a high rate of hypertension among patients with NL (*p* > 0.05). Although the results do not reach statistical significance, the finding that 51.5% of women and 66.7% of men with ulcerated NL lesions also had arterial hypertension is nonetheless notable. The study fails to demonstrate a possible correlation between NL and obesity; however, they mention the inability to obtain precise body weight data. Moreover, they raise the question of whether obesity could also precipitate the ulceration of the NL lesions, as 45.5% of women and 66.7% of men with ulcerated lesions were overweight [60] (Table 1).

**Table 1:** NL and associated comorbidities.

Author, Reference, Year of Publication	Type of Study	Studied Population	Comorbidities
Muller 1966 [64]	Retrospective Study	N = 171P NL+ N1 = 131 F (77%) N2 = 40 M (23%)	Patients with DM: N^1^ =111 P (64.9%) N1^1^ = 79 F (71%) vs. N2^1^ = 32 M (29%) Patients without DM: N^2^ = 60 P (35.1%) N1^2^ = 52 F (87%) vs. N2^2^ = 8 M (13%)
O’Toole EA 1999 [59]	Retrospective Study	N = 65 P NL+	Patients with DM: N^1^ = 7 P (11%) Patients with IGT: N^2^ = 2 P (5%) Patients with NIDDM: N^3^ = 1
De Silva BD 1999 [66]	Cross-Sectional Study	N = 1557 P (Children) DM1+	Patients with NL: N = 1 P (0.006%)
Erfurt-Berge 2012 [63]	Retrospective Study	N = 52 P NL+ N1 = 76.9% F N2 = 23.1% M	Patients with DM: N^1^ = 24 P (46%) Patients with thyroid disease: N^2^ = 7 P (13%) Patients with hypertension: N^3^ = 21 P (40%) Patients with obesity: N^4^ = 7 P (13%) Patients with dyslipidemia: N^5^ = 12 P (23%)
Erfurt-Berge 2015 [62]	Retrospective Study	N = 100 P NL+ N1 = 23 M N2 = 77 F	Patients with DM: N^1^ = 43 P N1^1^ = 12 M (27.9%) vs. N2^1^ = 31 F (72.1%) Patients with thyroid disease (autoimmune thyroiditis): N^2^ = 7 P N1^2^ = 1 M vs. N2^2^ = 6 F Patients with hypertension: N^3^ = 38 P N1^3^ = 10 M vs. N2^3^ = 28 F Patients with obesity: N^4^ = 18 P N1^4^ = 5 M vs. N2^4^ = 13 F Patients with dyslipidemia: N^5^ = 23 P N1^5^ = 6 M vs. N2^5^ = 17 F
Marcoval J. 2015 [60]	Retrospective Study	N = 35 P NL+ F = 29 M = 6 N1 = 23 DM+ (65.71%) N2 = 12 DM−	Patients with thyroid disease (hypothyroidism): N^1^ = 6 P N1^1^ = 4 P vs. N2^1^ = 2 P
Hammer 2016 [67]	Retrospective Study	N = 64 133 P DM1+ F = 47.7% M = 52.3% N1 = 161 P NL+ N2 = 63 972 NL−	Patients with dyslipidemia: N^1^ N1^1^= 53.4% vs. N2^1^ = 42.12% (*p* = 0.014) Patients with thyroid antibody positive: N^2^ N1^2^ = 24.8% vs. N2^2^ = 17.5% (*p* = 0.051) Patients with coeliac disease: N^3^ N1^3^ = 3.4% vs. N2^3^ = 1.0% (*p* = 0.0035)
Jockenhofer 2016 [74]	Retrospective Study	N = 262 P NL+ N1 = 166 F (63.3%) N2 = 96 M (36.6%)	Patients with DM: N^1^ = 90 P(34.4%) Patients with hypertension N^2^ = 24 P(9.2%) Patients with obesity N^3^ = 12 (4.6%) Patients with dyslipidemia N^4^ = 6 (2.3%)
Hashemi 2019 [72]	Retrospective Study	N = 236 P NL+ F = 200 M = 36 N1 = 138 P (58.5%) DM+ N2 = 96 P (41.5%) DM−	Patients with hypertension: N^1^ = 104 P N1^1^ = 74 P DM+ vs. N2^1^ = 30 P DM− (*p* < 0.001) Patients with dyslipidemia: N^2^ = 98 P N1^2^ = 69 vs. N2^2^ = 29 (*p* = 0.02) Patients with thyroid disease N^3^ = 56 P N1^3^ = 39 vs. N2^3^ = 17 (*p* = 0.4) Patients with obesity N^4^ = 95 N1^4^ = 59 vs. N2^4^ = 36 (*p* = 0.37)
Trihan 2020 [65]	Cross-Sectional Study	N = 213 P DM+ N1 = 6 NL+ (2.8%)	Patients with microvascular disease: N1^1^ = 9.7 (3.47–18.78) (*p* ≤ 0.001)
Erfurt-Berge 2022 [61]	Retrospective Study	N = 98 P NL+ N1 = 82 F (83.7%) N2 = 16 M (16.3%)	Patients with DM: N^1^ = 53 P (54%) N1^1^ = 33 F vs. N2^1^ = 20 M Patients with thyroid disease: N^2^ = 19 P (19.4%) (*p* < 0.05%) Patients with obesity: N^3^ = 36 P (36.7%) Patients with hypertension: N^4^ = 23 P (38.8%) (*p* > 0.05) Patients with hyperlipidemia: N^5^ = 14 P (14.3%)
Severson 2022 [73]	Retrospective Study	N = 328 P NL+ F = 282 (86.0%) M = 46 (14.0%) N1 = 167 P DM+ N2 = 131 P DM-	Patients with hypertension: N^1^ = 153 P N1^1^ = 97 (66%) vs. N2^1^ = 50 (42.2%) (*p* = 0.003) Patients with hyperlipidemia: N^2^ = 160 P N1^2^ = 103 (71.0%) vs. N2^2^ = 55 (51.4%) (*p* = 0.001) Patients with obesity: N^3^ = 70 P N1^3^ = 47 (58.0%) vs. N2^3^ = 22 (53.7%) Patients with thyroid disease: N^4^ = 92 P N1^4^ = 52 (37.1%) vs. N2^4^ = 37 (32.7%) (*p* = 0.466)
Vâță D 2023 [75]	Retrospective Study	N = 103 P DM+ N1 = 60 F (58%) N2 = 43 M (42%)	Patients with NL: N^1^ =7 P N1^1^ = 5 vs. N2^1^ = 2 (*p* = 0.466)

P: Patients; F: Females; M: Males; N: Number of patients; vs: versus; NL: Necrobiosis Lipoidica; DM: Diabetes Mellitus; IGT: Impaired glucose tolerance; NIDDM: Non-insulin-dependent diabetes mellitus.

**Table 2:** NL and DM complications.

Author, Reference, Year of Publication	Type of Study	Studied Population	Diabetes Complications
Boulton 1988 [76]	Cross-Sectional Study	N = 15 P N1 = 14 P DM1+ N2 = 1 P DM2+	Patients with retinopathy: N^1^ = 75% Patients with peripheral neuropathy: N^2^ = 18%
W F Kelly 1993 [69]	Case Control Study	N = 20 P DM+ N1 = 15 P NL+ N2 = 5 P NL−	Patients with retinopathy: N^1^ N1^1^ = 67% vs. N2^1^ = 27% (*p* = 0.009) Patients with proteinuria: N^2^ N1^2^ = 53% vs. N2^2^ = 17% (*p* = 0.006)
Verrotti 1995 [68]	Case Control Study	N = 58 DM1+ N1 = 18 P NL+ N2 = 40 P NL−	Patients with retinopathy: N^1^ N1^1^ = 12 (66.6%) vs. N2^1^ = 4 (10.0%) (*p* < 0.001) Patients with persistent microalbuminuria: N^2^ N1^2^ = 10 (55.5%) vs. N2^2^ = 5 (12.5%) (*p* < 0.001) Peripheral neuropathy: N^3^ N1^3^ = 2 (11.1%) vs. N2^3^ = 3 (7.5%) (NS)
Hammer 2016 [67]	Retrospective Study	N = 64 133 P DM1+ F = 47.7% M = 52.3% N1 = 161 P NL+ N2 = 63 972 NL−	Patients with retinopathy: N^1^ N1^1^ = 2% vs. N2^1^ = 1.1%; (*p* = 0.092) Patients with microalbuminuria:N^2^ N1^2^= 11.7% vs. N2^2^ = 9.6%; (*p* = 0.526)
Trihan 2020 [65]	Cross-Sectional Study	N = 213 P DM+ N1 = 77 Cutaneous signs of DM+ N2 = 136 Cutaneous signs of DM−	Patients with retinopathy: N^1^ N1^1^ = 6 (7.8%) vs. N2^1^ = (2.2%) Patients with nephropathy:N^2^ N1^2^ = 4 (5.2%) vs. N2^2^ = 7 (5.1%) Patients with peripheral artery disease: N^3^ N1^3^ = 34 (44.2%) vs. N2^3^ = 31 (22.8%) (*p* = 0.002)
Severson 2022 [73]	Retrospective Study	N = 328 P NL+ F = 282 (86.0%) M = 46 (14.0%) N1 = 131 DM− N2 = 167 DM+	Patients with retinopathy: N^1^ = 38 P N1^1^ = 0 (0%) vs. N2^1^ = 38 (22.8%) (*p* < 0.001) Patients with renal disease:N^2^ = 44 P N1^2^ = 39 (40.6%) vs. N2^2^ = 5 (5.7%) (*p* < 0.001) Patients with peripheral neuropathy: N^3^ = 68 P N1^3^ = 62 (37.1%) vs. N2^3^ = 6 (4.6%) (*p* < 0.001)

P: Patients; F: Females; M: Males; N: Number of patients; vs: versus; NL: Necrobiosis Lipoidica; DM: Diabetes Mellitus; DM1: Diabetes Mellitus type 1.

**Table 3:** NL and glycemic control.

Author, Reference, Year of Publication	Type of Study	Studied Population	Parameters
Dandona 1981 [77]	Cross-sectional Study	N = 22 P NL+ N1 = 9 P DM+ N2 = 11 P DM−	Glycosylated hemoglobin (%): N1 = 10.7 ± 2.5 vs. N2 = 6–6 ± 0–6 (*p* < 0.001)
W F Kelly 1993 [69]	Case Control Study	N = 20 P DM+ N1 = 15 P NL+ N2 = 5 P NL−	Glycosylated haemoglobin (%): N1 = 8.6 ± 1.5 vs. N2 = 7.4 ± 1.7 (*p* = 0.02)
Ryan 2001 [71]	Prospective Study	N = 12 P DM1+ N1 = 1 P NL+	Patients with amelioration of NL after islet transplantation: N^1^ N1^1^ = 1 P
Souza 2009 [70]	Retrospective Study	N = 15 P N1 = 11 P PT, PT + KT (5 P NL+) N2 = 4 P KT (1 P NL+)	Patients with remission of NL after transplantation: N^1^ = 5 P N1^1^ = 5 vs. N2^1^ = 0

P: Patients; F: Females; M: Males; N: Number of patients; vs: versus; NL: Necrobiosis Lipoidica; DM: Diabetes Mellitus; DM1: Diabetes Mellitus type 1; PT: Pancreatic transplant; PT + KT: Pancreatic transplant and kidney transplant; KT: Kidney transplant.

## 4. Discussion

### 4.1. Etiopathogenesis

The etiology of NL has been a subject of speculation for an extended period of time, although a definitive conclusion remains elusive. The most widespread theories are as follows: vascular impairment, anomalies in collagen formation, microangiopathic alterations that result in collagen degradation, immune complex deposition, impairment in neutrophil migration, and genetic implications. Conflicting studies have persisted, leading to ongoing controversy around the cause and pathophysiology of NL.

#### 4.1.1. Vascular Impairment

Multiple studies have focused on investigating diabetic microangiopathy as the primary etiological theory because of the significant association between DM and NL. The vascular abnormalities observed in NL are similar to the modifications from retinopathy and nephropathy. The thickening of the vessels’ walls, fibrosis, and endothelial growth resulting in occlusion in the deeper dermis are the most frequent vascular abnormalities seen in NL lesions. The connection with DM has strengthened the hypothesis that microangiopathy results from glycoprotein accumulation in the blood vessels’ walls [78]. An immunohistochemical study reveals the upregulation of Glut-1 receptors in fibroblasts, suggesting a potential association with reduced blood flow. The precise process responsible for the receptors’ enhanced presence is still up for discussion [58]. Also, reduced oxygen tensions in NL locations have been shown as evidence in support for this microangiopathic theory [79]. In a study including 10 idiopathic cases of NL, researchers observed a decrease in oxygen tension, but an increase in laser Doppler fluxes surrounding NL lesions when exposed to hyperemic stimuli [79]. However, a Doppler flowmetry study that revealed higher blood flow in NL areas compared to uninvolved skin raised doubts about the validity of this claim [80].

#### 4.1.2. Collagen Anomalies

Another hypothesis centers on the presence of atypical collagen in patients affected by NL. One well-established cause of diabetic end-organ damage is defective, aberrant collagen fibrils. Collagen crosslinking is increased in diabetics because of the elevated lysyl oxidase levels. The increased collagen crosslinking may explain the thicker basement membrane in NL [81,82]. One notable observation is the absence of cross striations; moreover, certain cases even indicate a total loss of elastin and collagen [83]. There have also been reports of anti-collagen antibodies in NL and other granulomatosis patients; however, the levels were similar to those from the control group in Evans et al.’s study [84].

#### 4.1.3. Immune Complex Deposition

Immunofluorescence investigations have demonstrated the presence of fibrin, IgM, and C3 deposits at the dermo-epidermal junction of blood vessels. Furthermore, antibody-mediated vasculitis has the potential to induce structural alterations in the vasculature, ultimately causing the blockage of dermal vessels and subsequent necrobiosis [85].

#### 4.1.4. Impairment in Neutrophil Migration

In the initial phases of the disease, there is an observable presence of an inflammatory infiltrate that is predominantly composed of neutrophils [86]. The process of granuloma formation is believed to arise from impaired neutrophil movement, which leads to the macrophages assuming the role of neutrophils. Ultimately, the macrophages accumulate, resulting in the development of granulomas [81].

#### 4.1.5. Genetic Implications

Occasional cases with familial history of NL have been documented. NL was observed in some patients in the absence of DM or glucose intolerance and concurrent with DM [87,88,89]. The genetic hypothesis remains debatable [90]. A case-control study proved that diabetic patients with NL have a decreased frequency of HLA-A2, compared to diabetic patients without NL. However, the lack of significant variations between patients with and without necrobiosis supports the idea that vascular and/or metabolic variables, rather than genetic ones, play a more important role in the etiology of NL [91].

Additional theories support that trauma, inflammation, chronic venous insufficiency, and metabolic alterations could cause NL [81]. Multiple case reports mention that chronic venous insufficiency, as well as hypercholesterolemia, could trigger NL [92,93].

### 4.2. Comorbidities

#### 4.2.1. Diabetes Mellitus

Although NL is quite rare among diabetic patients (0.3% to 2.8%), DM remains the most frequently associated comorbidity in patients affected by NL. There is a large variety in the prevalence rates of DM among NL patients, from 11 to 65.71 percent [59,60]. It is important to bear in mind that both a familial predisposition and a prospective diagnosis of diabetes may constitute significant factors to be taken into account in the epidemiological analysis of NL [64]. The presence of different statistical data obtained from several studies have resulted in ongoing disagreement regarding the precise prevalence rates of NL in individuals with DM.

It has been shown that NL may manifest before DM is diagnosed [59]. The mean age of onset is approximately 3rd-4th decade. Insulin-dependent individuals with T1DM exhibit a higher likelihood of developing NL at a younger age, with an average onset occurring at 22 years. In contrast, individuals with T2DM and those without DM tend to experience the onset of NL at an average age of 49 years [59]. A higher prevalence has been observed among females, with a mean ratio of females/males from 3:1 to 5:1 [72]. The role of estrogen in the etiology of NL lesions has been discussed, although the specific mechanism behind this relationship is still not fully understood. The literature suggests that estrogen has been found to benefit skin vascularization and enhance collagen content and skin quality [94]. Based on the information provided, it may be concluded that estrogen exerts a preventive rather than promotive effect on the development of NL. Nevertheless, estrogen demonstrates the potential for an elevated susceptibility to immunological disorders. In a 1995 study, sera from individuals with NL showed elevated natural autoantibody activity against cytoskeleton proteins [95].

Whether NL is more common in type 1 diabetes or type 2 diabetes is debatable, as the findings have exhibited significant variability. This notable disparity may be attributed, in part, to the greater occurrence of impaired glucose tolerance in North America compared to Europe (54% vs. 36%) [72]. However, it underscores the significance of taking into account NL in individuals with any form of DM [72]. There have been studies documenting its prevalence in pediatric populations; however, it is extremely rare [66,68].

There is also ongoing conflicting evidence over the potential impact of enhanced glycemic control on the clinical progression of NL. According to Lowitt and Dover, there is evidence suggesting no correlation between NL and glycemic control [5].

Multiple studies have shown no discernible variation in glycosylated hemoglobin values between NL-positive and NL-negative patients [68,69,96,97]. According to the findings of Reid et al., it was determined that there is no significant impact of glucose control on the occurrence or advancement of NL [2]. However, a study conducted by Cohen et al. has contested this statement [98]. They carried out a comprehensive review reporting that patients who achieved better glycemic control experienced improvements in the progression of NL lesions [98]. Moreover, two studies proved that after pancreatic transplant, and after islet transplant, respectively, NL lesions are more prone to resolution [70,71]. Mistry et al., in their analysis of data derived from case studies and case reports, suggest that enhanced glycemic control has the potential to result in the resolution of NL in individuals with concurrent DM [99]. Hammer et al. also reported a positive association between elevated levels of HbA1c, extended duration of diabetes, and the occurrence of NL [67]. Thus, it is recommended that individuals diagnosed with NL who do not have a confirmed diagnosis of DM undergo screening for either a fasting blood glucose concentration or HbA1c level at the time of their initial medical presentation, due to the widely recognized correlation with diabetes.

One notable observation is the heightened prevalence of retinopathy and systemic microvascular disease in individuals diagnosed with both NL and DM. Thus, these patients should be closely monitored for any potential organ function damage [69]. This correlation is also observed in pediatric populations [68]. A notably elevated occurrence of persistent microalbuminuria and retinopathy was observed among the cohort of children and adolescents diagnosed with Type 1 DM and NL [68]. Trihan et al. also support a strong association between NL and microvascular disease [65].

Hence, it is plausible that NL may serve as a prognostic marker in diabetic patients.

#### 4.2.2. Thyroid Disease

Thyroid disorders, such as Graves disease and Hashimoto disease, have been documented in certain patients with NL lesions on the lower extremities [10,29,100]. Erfurt-Berge et al. reported in their 2012 study, that from a group of 52 participants, 7 individuals were found to have thyroid disease [63]. There is currently no established causal association between the two disorders; nonetheless, there has been speculation regarding the presence of autoantibodies or deposition of autoimmune complexes [29,100]. In both cases, there is growing evidence suggesting that NL may be triggered by an immunological mechanism, in which either an immune complex disease or autoantibodies are directed against vessel wall tissue antigens [100]. This theory could be supported by the immune complex deposition hypothesis of NL etiopathogenesis, explained above. Moreover, the greater correlation between NL and thyroid disease in T1DM patients as opposed to T2DM patients further suggests an immune-mediated mechanism. Marcoval et al. observed in their study that 17% of the participants (6 patients) exhibited hypothyroidism. It is important to note that this correlation could potentially be attributed to the simultaneous presence of autoimmune thyroid disease and T1DM, as 4 out of the 6 patients presented with T1DM [60]. Borgia et al. describe a case of Hashimoto’s thyroiditis in an NL patient, associated with high titers of both ANA and ASMA. The patient had no other autoimmune pathologies or liver disease [100]. Nevertheless, the current understanding of the prevalence and clinical importance of non-organ-specific autoantibodies in the serum of individuals with autoimmune thyroid disorders remains limited [100]. In Hammer et al.’s study, a total of 40 819 individuals diagnosed with T1DM were examined for the presence of thyroid antibodies. Among this group, 18.5% were found to have tested positive for these antibodies. Additionally, out of the 97 patients who had both T1DM and NL, 32% were found to be positive for thyroid antibodies. Nevertheless, these results did not demonstrate statistical significance following adjustment for confounding variables [67]. This study is the first to establish a correlation between NL and celiac disease in individuals diagnosed with T1DM. It was found that the occurrence of histologically confirmed coeliac disease in individuals with T1DM was 1.0%, whereas it increased to 3.4% when NL was also present. This discovery can be taken as evidence of immune changes that contribute to the development of both NL and celiac disease [67].

Thus, with the link to thyroid disease, medical professionals should look for indications of thyroid dysfunction in patients’ history, as well as during physical examination. If these are found, they should also consider further testing including TSH levels and thyroid antibodies.

#### 4.2.3. Metabolic Syndrome

Individuals who have both T1DM and NL have been diagnosed with diabetes for a longer period (6.24 vs. 5.11 years) and sooner (8.16 vs. 8.85 years) than those who do not have the NL. Hence, it is suggested that the metabolic implications of elevated blood glucose levels may contribute to the development of NL [67].

Multiple studies suggest that the occurrence of ulcers on NL lesions might be associated with the presence of hypertension, hyperlipidemia, and obesity. In the Erfurt-Berge et al. study, while the statistical significance of the data was not achieved, it was worth noting that a considerable proportion of individuals with ulcerated NL lesions, specifically 51.5% of women and 66.7% of men, also exhibited arterial hypertension. Additionally, the authors propose that obesity may contribute to the development of ulceration in NL lesions, given that 45.5% of women and 66.7% of men with ulcerated lesions were classified as overweight [61]. In the Franklin et al. case report series, higher rates were provided. A total of 60% of the patients diagnosed with ulcerated necrobiosis lipoidica had comorbidities including arterial hypertension, obesity, smoking, and hypercholesterolemia [1]. This correlation can be accounted for by the fact that optimal fat and carbohydrate metabolism is unattainable in even medically well-controlled forms of DM. Additionally, this phenomenon contributes to the development of diabetic microangiopathy and arteriosclerosis, subsequently leading to the occurrence of arterial hypertension. Hence, the explicable nature of a possible strong correlation between NL and other metabolic syndrome-related disorders within this study becomes more evident [1].

In a more recent study, Hashemi et al. also revealed a high proportion of patients with hypertension (45.2%) and dyslipidemia (43.6%). The observed link between obesity and NL, found at comparable frequencies among individuals with and without diabetes (52.2% vs. 50.7%), suggests that metabolic processes beyond blood glucose may play a potential role in the pathophysiological features of this condition [72].

Jockenhöfer et al. reported in their study that arterial hypertension was the second most common secondary diagnosis besides DM. Other secondary diagnoses consisted of obesity (4.6%) and dyslipidemia (2.3%) [74]. Furthermore, an immunohistochemical investigation has shown findings supporting a distinct extracellular adipophilin expression pattern in regions displaying collagen abnormalities. This observation has the potential to enhance the ability to distinguish between NL, granuloma annulare, and sarcoidosis [101]. Adipophilin, also referred to as perilipin 2 or adipose differentiation-related protein (ADRP), is a protein that exhibits accumulation within human adipocytes. Moreover, under conditions of lipid excess, adipophilin can also be observed on the surface of lipid droplets in various metabolically active cell lines, including fibroblasts, endothelial cells, epithelial cells, and others. Consequently, it could potentially serve as an indicator for related disorders [101,102]. Concerning the previously indicated elements of the particular adipophilin expression pattern, it is plausible that obesity and hyperlipidemia, which are connected to lipid buildup, could potentially be correlated with NL [74]. A case report has documented the remission of ulcerated NL in a patient with morbid obesity, following bariatric surgery [103].

### 4.3. Therapeutic Barriers

NL therapy can be difficult, frequently requiring several modalities before a response is seen. The optimization of glucose levels should be considered a primary objective in the comprehensive management of patients diagnosed with DM [17]. Nonetheless, it is crucial to recognize that glucose optimization should be pursued in conjunction with other therapeutic interventions [81]. Equally crucial for reducing complications and facilitating the process of healing are supplementary adjustments in lifestyle, such as the cessation of smoking and the avoidance of trauma [2,17,69]. The occurrence of venous illness or lymphedema might potentially worsen NL, particularly in cases where ulcers are present [17,92,104]. Compression treatment effectively manages edema and facilitates the healing process in individuals with these conditions. Furthermore, it is possible to protect nonulcerated lesions on the lower legs by utilizing support stockings as a preventive measure against trauma and the occurrence of the Koebner phenomenon. Adherence to appropriate wound care guidelines is crucial in cases where ulcerations are evident. Moreover, it is vital to identify infections rapidly. Systemic antibiotic therapy is required for the treatment of deep infections, but superficial infections can be effectively managed by utilizing topical antiseptics [17].

Corticosteroids, namely topical, intralesional, or sometimes systemic, currently serve as the primary therapeutic approach [15,16,105,106]. Other treatment options include PUVA, photodynamic therapy, immunomodulators (Methotrexate, Mycophenolate mofetil), antimalarials (hydroxychloroquine and chloroquine), fumaric acid esters, cutaneous blood flow modulators (aspirin, dipyridamole, pentoxifylline, ticlopidine), biologics (anti-TNF alpha, Ustekinumab), cyclosporine, topical tretinoin, thalidomide, and surgery with excision followed by skin grafting [25,107,108,109,110,111,112,113,114,115,116,117]. Several recent case reports have proven the efficacy of Janus kinase inhibitors (JAK) such as ruxolitinib, baricitinib, and tofacitinib [118,119,120,121,122]. JAKs have proven to be effective in the treatment of inflammatory diseases. These molecules inhibit the activity of the JAK family of enzymes’ activity, which are tyrosine kinase enzymes responsible for transmitting signals from surface receptors to the nucleus. JAKs regulate more than 50 cytokines and growth factors [121].

As mentioned above, the first-line therapy in NL management is represented by corticosteroids, topical, intralesional, or even systemic. Patients with DM should have their topical steroid use closely managed to avoid glucose dysregulation, especially when vast surface areas are involved. Atrophic areas should not receive application of corticosteroids due to their ineffectiveness and potential to exacerbate atrophy. Instead, steroids should be given to their active borders to lessen inflammation and stop lesion growth [2]. Systemic corticosteroids are efficacious in the treatment of quickly progressive lesions. However, it is essential to note that the development of hyperglycemia and hypertension often accompanies their use. These adverse effects can be particularly troublesome in individuals with DM [123]. Systemic corticosteroids might be contraindicated in DM patients, depending on the severity of the illness. A study demonstrates that the use of corticosteroids in short-course therapy is an efficient method to stop the advancement, and may offer long-term defense against the reappearance of NL. Considering that inflammation and granuloma formation exhibit favorable responses while atrophy does not, it is advisable to administer medication promptly during the disease’s progression to achieve optimal benefits for the patient. The medication showed excellent tolerability and did not present any complications, even among DM patients [82].

On the other hand, one notable finding in Hammer et al.’s study was the observation of a significant increase in insulin requirement, regardless of body weight among patients with T1DM and NL, as compared to those without NL. One possible interpretation for this discovery could be the increased prevalence of traditional DM treatment among those with NL compared to those without NL (14.4% vs. 8.0%). The conventional treatment approach for diabetes was characterized by the administration of one to three daily injections, typically involving greater insulin doses and resulting in poorer glycemic control compared to intensive insulin therapy and insulin pump therapy. Nevertheless, the observed disparity in insulin dosage per kilogram of body weight between the two cohorts was validated (*p* < 0.0001) [67]. The insulin dose measures insulin resistance in children and adolescents. Multiple studies have posited that insulin resistance plays a role in the pathogenesis of microvascular complications in T2DM [67,124]. Hence, the etiological hypotheses regarding NL encompass insulin resistance and endothelial dysfunction, potentially exhibiting a correlation with the aforementioned assertion.

## 5. Conclusions

This review emphasizes the intricate associations between NL and various comorbidities, including DM, thyroid disorders, and metabolic syndrome. Further research is warranted to elucidate the complex interplay between NL and these conditions, providing insights into potential shared pathophysiologic mechanisms and optimizing patient care. Managing a patient with Necrobiosis Lipoidica remains a challenge, frequently requiring several modalities before observing a response. Optimizing glucose levels should be considered a primary objective in cases where NL is associated with DM.

## Data Availability

Not applicable.
